# Removal of Microplastics from Laundry Wastewater Using Coagulation and Membrane Combination: A Laboratory-Scale Study

**DOI:** 10.3390/membranes15020047

**Published:** 2025-02-04

**Authors:** Thi Trang Luu, Dai Quyet Truong, Van Nam Nguyen, Sanghyun Jeong, Thi Thu Trang Nguyen, Van Manh Do, Saravanamuthu Vigneswaran, Tien Vinh Nguyen

**Affiliations:** 1 Faculty of Engineering and IT, University of Technology Sydney (UTS), Sydney, NSW 2007, Australia; thitrang.luu@student.uts.edu.au (T.T.L.); daiquyet.truong@student.uts.edu.au (D.Q.T.); s.vigneswaran@uts.edu.au (S.V.); 2 Faculty of Urban Infrastructure and Environment Engineering, Hanoi Architectural University, Hanoi 100000, Vietnam; namnv@hau.edu.vn; 3 School of Civil and Environmental Engineering, Pusan National University (PNU), Busan 46241, Republic of Korea; sh.jeong@pusan.ac.kr; 4 Institute of Science and Technology for Energy and Environment, Vietnam Academy of Science and Technology (VAST), Hanoi 100000, Vietnam; nguyenthithutrang@istee.vast.vn (T.T.T.N.); dovanmanh@istee.vast.vn (V.M.D.)

**Keywords:** microplastics, occurrence, laundry wastewater, coagulation, membrane filtration

## Abstract

Microplastic (MP) pollution has recently emerged as a critical global environmental issue. Laundry wastewater is a significant contributor to MP pollution, containing high concentrations of MPs. Although coagulation has recently been widely applied to remove MPs from such wastewater, its efficiency remains poor, and the removal mechanisms are not yet fully elucidated. In this study, the occurrence and characteristics of MPs in raw domestic laundry wastewater were investigated. The coagulation process was combined with ultrafiltration (UF) membrane filtration to enhance MP removal. The results showed that the concentrations of MPs in laundry wastewater ranged from 9000 to 11,000 particles/L, with fibrous particles constituting the majority (42.6%) and polyester accounting for 68.2% of detected MPs. Using aluminium chloride and ferric chloride as coagulants, maximum removal efficiencies of 91.7 and 98.3% were achieved, respectively. Mechanistic analysis revealed that charge neutralization played a dominant role during coagulation. Fourier transform infrared spectroscopy further demonstrated the formation of new functional groups, substituted benzene rings, and the presence of Fe-O and Al-O bonds, indicating the interaction between MPs and coagulants. Furthermore, the UF membrane was used to remove fibrous MPs and MPs with low densities. These MPs had not been removed with pre-coagulation. The removal efficiency of these MPs reached 96 ± 2%, reducing their concentration to only 60 particles/L in the UF permeate. These findings highlight the synergistic potential of coagulation and UF membrane filtration for effective MP removal and provide a valuable reference for advancing wastewater treatment technologies targeting MP pollution.

## 1. Introduction

The term “microplastics” (MPs) was first introduced by Thompson et al. in 2004. They defined MPs as plastics smaller than 5 mm, although most are smaller than 500 μm [1,2]. MPs can arise from the disintegration of plastic particles like pellets used in cosmetics or industrial processes, scrubbers, polymer fragments, or pieces of baggage [3]. They come in two forms: primary and secondary. Primary MPs are specifically produced in small sizes and are commonly used as scrubbers or industrial pellets in toiletries and beauty products [2]. Meanwhile, secondary MPs are tiny plastic fragments that result from the breakdown of larger plastic debris [4]. MPs are made up of 14 different polymers, with polyethylene, polypropylene, and polystyrene being the three most common [2,5]. Previous research has demonstrated that chemical pollutants such as nonylphenol and triclosan can attach to the surfaces of MPs. When aquatic organisms ingest these MPs, the pollutants are absorbed and accumulate in their bodies [6,7,8]. There is significant evidence suggesting that MPs could exert harmful effects on people’s health through both exposure and toxicity pathways [9,10,11]. To address the issue of MPs, scientists, policymakers, and funders are crucial in forming international networks and working together to tackle this global challenge [12].

Laundry activities have been identified as a major source of MPs, with concentrations ranging from 124 to 308 mg/kg of fabric, largely depending on the type of garment. Interestingly, a considerable number of cellulosic microfibres were found when the laundry garments were made from a polyester/cellulose blend [13,14,15]. It was estimated that a typical 5 kg wash load could release more than 6,000,000 MPs, varying based on the detergent type [13]. Studies by De Falco et al. (2018) revealed that using a conditioner with a lint collection bag reduced microfibre release by more than 35%. The bag captured MPs larger than 1000 μm but not those smaller than 500 μm [13]. When it comes to removing MPs from laundry wastewater, the coagulation method’s removal efficiency ranged from 86% to 96%, depending on the MP size, with smaller microfibres showing lower removal efficiency [16]. Meanwhile, in the treatment of wastewater from a washing machine designed to clean industrial tents, the microfiltration membrane revealed a greater diminishment in permeability (95%) compared to the ultrafiltration membrane (37%) [17,18]. Given the global rise in the production and use of synthetic textiles, identifying the characteristics of MPs and effective methods for their removal from laundry wastewater is a major challenge. This is due to the limited number of studies and frequent contradictions in existing research [19,20,21].

Recently, methods for removing MPs have received widespread attention and research. Coagulation and membrane filtration are also common methods, each with its own advantages and disadvantages. In this study, we combined these two methods to achieve the most effective treatment of MPs in laundry wastewater. We first studied the characteristics and distribution of MPs in wastewater. Then, coagulation utilizing different coagulants with various concentrations was applied as a primary treatment to remove MPs in order to identify a suitable coagulant and its dosage. At the same time, the mechanism and influential factors of the coagulation process were explored. Finally, membrane ultrafiltration was used as a secondary treatment step after coagulation, with the aim of increasing the overall system performance and reducing membrane fouling.

## 2. Materials and Methods

### 2.1. Chemicals and Materials

Real laundry wastewater samples were collected from a residential washing machine (Bosch Serie 4 WLG24225, Stuttgart, Germany) after two wash/spin cycles. The laundry involved 5 kg of red T-shirts and 45 mL of liquid detergent applied at the manufacturer’s recommended dosage. The washing process utilized the synthetic clothes program at 40 °C and 1200 rpm, with a 30 min quick wash mode. Wastewater was sampled directly from the washing machine’s drainpipe after two rinses, specifically at 12 min and 18 min from the start of operation. The laundry wastewater samples were then directly transported to the sample storage room in the laboratory and kept at a temperature of 4 °C, and the subsequent experiments were carried out in the laboratory.

To ensure the laundry wastewater consisted of a high concentration of MPs, additional small PE and PET particles were introduced to the raw household laundry wastewater. This adjustment resulted in solutions containing 100 mg/L of PE and PET. The added PE and PET particles had sizes ranging from approximately 20 to 1000 µm, with densities of 0.91 g/cm^3^ and 1.3 g/cm^3^, respectively. They mainly had a granule shape.

For the coagulation tests, aluminium chloride hexahydrate (ACH) and iron (III) chloride (FeCl_3_) were used, both purchased from Sigma Aldrich (St. Louis, MO, USA) with an analytical reagent grade and a purity of 99%. Stock solutions of 500 mg/L were prepared by dissolving 0.5 g of each coagulant in 100 mL of deionized (DI) water. These stock solutions were stored in the dark at 4 °C prior to use in the experiments.

For the membrane test, the polyethersulfone (PES, MK series, Synder Filtration™, Gladwyne, PA, USA) ultrafiltration (UF) membrane used has an MWCO of 30 kDa and a pH range of 1–11 and is classified as a hydrophilic membrane. The flux of the membrane used ranges from 169 to 260 GFD/60 psi. The membrane was activated by soaking in ethanol for 10 min, followed by rinsing in DI water. It was then stored in refrigerated conditions overnight before use. The membrane’s separation performance was tested with a lab-scale UF system (Cheon Ha Heavy Industries Co., Ltd., Gwangju, South Korea), which includes a cross-flow cell with an effective membrane area of 68 cm^2^.

### 2.2. Sample Preparation and Characterization of MPs

Initially, 50 mL of laundry wastewater was supplemented with 50 mL of 30% H_2_O_2_ to remove biological organic matter, as described in [22]. The mixture was then heated to 100 °C for 2 h for the digestion process. A density separation step was unnecessary due to the intrinsic properties of the laundry wastewater. The solution containing MPs was subsequently filtered through an Anodisc filter (pore size: 0.2 µm) using a vacuum pump. The Anodisc filters with attached MPs were kept in covered glass Petri dishes for further characterization. To identify MP characteristics, a Spectrum 3/Spotlight 400 Fourier transform infrared (FTIR) spectrometer (Waltham, MA, USA) equipped with a microscope was employed for the observation, analysis, and imaging of particles on the Anodisc filters. This equipment is able to detect MPs within the wavelength range of 4000–400 cm^−1^. Particle counts were performed directly on the Anodisc filters, which were divided into 1 cm × 1 cm squares using the integrated microscope of the FTIR spectrometer.

The microscope was further used to quantify the total number of MP particles before and after the coagulation and membrane filtration tests. Additionally, raw household laundry wastewater collected from the domestic washing machine was examined for its MP properties. Precipitated flocs from the coagulation tests were oven-dried for subsequent FTIR analysis.

### 2.3. Coagulation and Membrane Filtration Experiments

The Jar Test was first used for the coagulation experiment. Each 1 L glass beaker contained 500 mL of high-strength laundry wastewater prepared by methods stated in the previous section. Coagulant doses of 0 mg/L, 30 mg/L, 60 mg/L, 90 mg/L, and 120 mg/L (corresponding to Al^+3^ and Fe^+3^ contents of 0 Al^+3^ mg/L, 3.35 mg Al^+3^/L, 6.71 mg Al^+3^/L, 10.06 mg Al^+3^/L, 13.42 Al^+3^ mg/L, and 0 Fe^+3^ mg/L, 6.21 mg Fe^+3^/L, 12.42 Fe^+3^ mg/L, 18.63 Fe^+3^ mg/L, and 24.84 mg Fe^+3^/L, respectively) were used in the Jar Test experiments. The stirring speed was first set at 300 rpm for 1 min and then reduced to 100 rpm for 15 min, followed by a 30 min sedimentation period. After coagulation and sedimentation, the supernatant was collected and filtered through a 0.02 µm Ano-disc inorganic filter membrane (obtained from Whatman, Marlborough, MA, USA, 25 mm Ø) for analysing filtered water. The flocs were also stored for the purpose of characterization. The zeta potentials, pH, and turbidity of the laundry wastewater with high MP concentrations during coagulation were measured using a zeta potential Zetasizer nano instrument (Malvern, UK), pH meter (HQ40d, Hach, Loveland, CO, USA), and portable turbidimeter (2100Q IS, Hach), respectively. Based on the coagulation study, the coagulant dosage that helped to achieve the highest MP treatment efficiency was selected as the optimal dosage.

In this study, the performance of UF in MP retention was investigated. The effect of the UF membrane on MP retention at the laboratory scale was recently examined by Luogo et al. [17]. In their study, the authors explored the effectiveness of using UF and MF membranes in removing contaminants from laundry wastewater. Their findings highlighted that the UF membrane was more efficient than MF in eliminating MPs from the wastewater. This was due to UF’s finer pores in the selective layer compared to those of the MF membrane [17]. Consequently, the UF membrane was chosen in this study.

In the membrane filtration system, the influent for the membrane system was the effluent obtained from the coagulation process at the optimal dosage of the coagulant. Here, the UF membrane pilot system (Cheon Ha Heavy Industries) was tested with an initial pressure of 2 bars. The efficiency of retention (by membranes) or removal (by coagulation) was assessed based on the reduction in MP concentration after the treatment of the MP concentration in the influent of the respective treatment process, as indicated in the Equation below:

E_retention/removal_ = (1 − C_MPs_effluent_/C_MPs_feed_) × 100% (1)

where C_MPs_feed_ represents the MP concentration prior to treatment, and C_MPs_effluent_ indicates the MP concentration following treatment.

### 2.4. Quality Control

To prevent the contamination of MPs from the surrounding environment, several strict protocols were adhered to during sampling and analysis [23]. These included cleaning the work area with alcohol prior to the procedure and using only glass or metal equipment and containers for sampling and analysis to ensure minimum contact with plastic materials to prevent MPs’ pollution. All experiments were implemented in triplicate, and the average values from three replicate samples were used to investigate the presence of MPs. Data are presented as mean ± standard deviation (SD).

## 3. Results and Discussion

### 3.1. Occurrence and Distribution of MPs in Raw Domestic Laundry Wastewater

The concentration of MPs in the laundry washing wastewater analysed in this study ranged from 9000 to 11,000 particles/L. The highest MP concentration and turbidity were observed in the last rinse effluent, while the lowest concentrations were detected in the first rinse effluent. Similarly, the pH measured during the second rinse was lower than that during the first rinse, which could be attributed to a reduction in detergent concentration leading to a pH increase. Le et al. (2022) reported that factors such as temperature, fabric types, detergents, and washing methods can significantly influence the release of MPs into wastewater during laundry processes [14].

**Figure 1:**
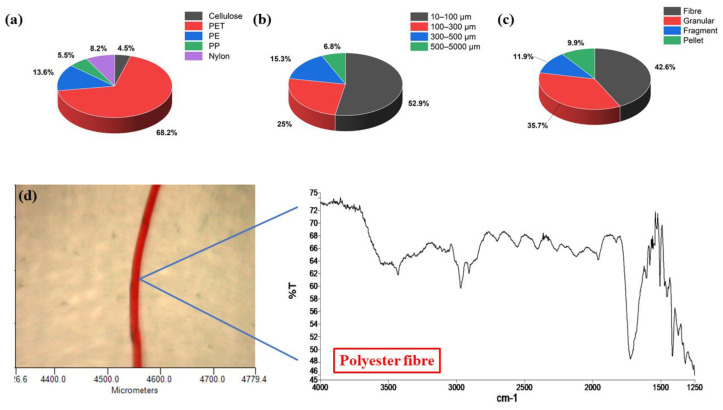
Occurrence and distribution of MPs in the raw domestic laundry wastewater. (**a**) Polymer types of MPs; (**b**) MP sizes; (**c**) MP shapes; (**d**) spectrum, shape, and colour of a typical PET fibre.

As shown in Figure 1, MPs were classified based on their polymer types, sizes, and shapes. The MPs detected in this study predominantly consisted of polyethylene terephthalate (PET), polyethylene (PE), polypropylene (PP), polystyrene (PS), nylon, and cellulose. PET was the most abundant polymer type, accounting for 68.2% of the total MPs, followed by PE at 13.6% (Figure 1a). Other synthetic polymers collectively contributed 18.2%, specifically PP, nylon, and cellulose, which made up 5.5%, 8.2%, and 4.5% of the total detected MPs, respectively. These findings align with results from previous studies [14,20,24]. MP sizes in the laundry wastewater were grouped into four categories: 10–100 μm, 100–300 μm, 300–500 μm, and 500–5000 μm. Figure 1b summarizes the distribution of MPs by size. It was observed that MPs in the 10–100 μm size range contributed 52.9% of the total MPs, whereas those in the larger-size 500–5000 μm range only made up 6.8% of the MPs in the laundry wastewater. In terms of morphology, fibrous particles were the most common, making up 42.6% of the total MPs, followed by granular particles at 35.7%. In contrast, fragmented and pellet-shaped microplastics made up a small proportion of laundry wastewater, accounting for only 11.9% and 9.9%, respectively (Figure 1c). These findings align with previous studies that have identified laundry wastewater as a primary source of microfibres in aquatic environments [19].

### 3.2. Performance of ACH and FeCl_3_ Coagulation for PET and PE Removal from Laundry Wastewater

#### 3.2.1. Turbidity

**Figure 2:**
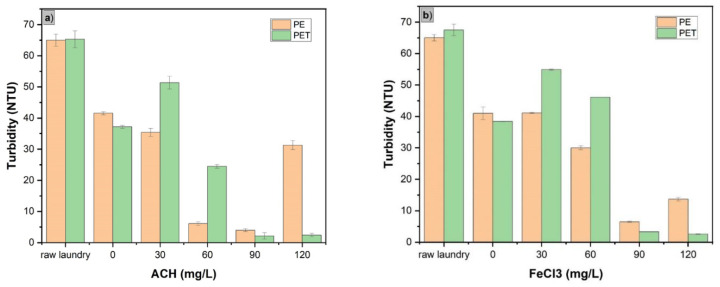
Turbidity of high-strength MP laundry wastewater at various coagulant dosages: (**a**) ACH and (**b**) FeCl_3_.

Jar Test experiments led to a significant reduction in the turbidity of laundry wastewater containing high-strength PE, and polyester PET was notably reduced. When using ACH as the coagulant, the decline in turbidity was more pronounced in wastewater with a high PET concentration compared to that with a high PE concentration. Specifically, Figure 2a shows that with an optimal coagulant dosage of 90 mg/L, the turbidity of wastewater with high-strength PE and PET was reduced from 65 NTU to 4 NTU and 2 NTU, respectively. This trend aligns with some previous studies [25,26] and is explained by the fact that PE, having a lower density than PET, settles less effectively, leading to lower coagulation treatment efficiency compared to PET. When the ACH dosage surpassed 90 mg/L, the efficiency in removing PET and PE either remained constant or decreased. With an ACH dosage of 90 mg/L, after coagulation, the turbidity of the laundry wastewater containing PE and PET MPs was 5 and 2 NTU, respectively. When the coagulation dosage increased to 120 mg/L, the turbidity values were 32 and 3 NTU, respectively. This could be due to excessive coagulant causing flocs to become loose and prone to breaking, which reduces coagulation effectiveness. A similar trend was observed with FeCl_3_ coagulation. As seen in Figure 2b, the removal efficiencies of PET and PE also initially increased when the FeCl_3_ dosage was elevated, whereas they remained nearly stable when the dosage continued to increase from 90 mg/L to 120 mg/L.

In summary, based on turbidity criteria, a dosage of 90 mg/L is ideal for both ACH and FeCl_3_ coagulants. However, ACH is more effective than FeCl_3_ in removing MPs from laundry wastewater. MPs of the PE type are also more difficult to remove compared to PET MPs due to the lower density of PE. However, evaluating the ability of ACH and FeCl_3_ coagulation to remove PET and PE based on turbidity is not very accurate, since there are many other components, such as organic particles and soluble compounds in laundry wastewater, that affect turbidity besides MPs [27]. Therefore, in the next section, the results of the quantity of MP particles before and after coagulation experiments are discussed.

#### 3.2.2. MP Particles

**Figure 3:**
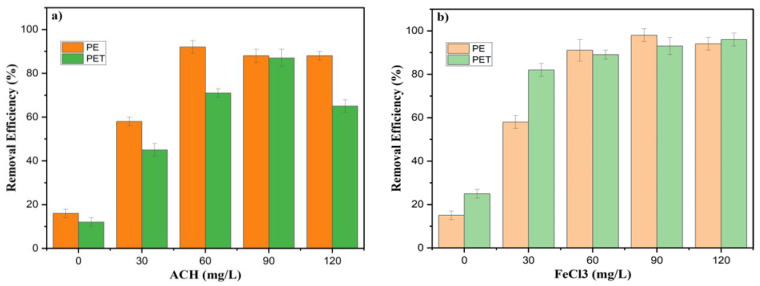
Removal efficiencies of PS and PE MPs at various coagulant dosages of PAC and FeCl_3_: (**a**) ACH and (**b**) FeCl_3_; [PET]_o_ = 100 mg/L, [PE]_o_ = 100 mg/L.

In order to further explore the removal performance of ACH and FeCl_3_ for PET and PE microplastics, the effects of different dosages were investigated. The removal efficiency of PET and PE was determined with different coagulants and doses. The removal efficiencies of PET and PE at different coagulant dosages are shown in Figure 3. In the absence of a coagulant, the removal efficiency of PE was only 12.15%, while that of PET was higher, 25.47%, which is linked to different densities. It is easier for PET to settle because of its higher density. Here, the removal of MPs without adding a coagulant can be explained by the fact that the laundry wastewater itself contains detergent, which acts as a coagulant, thus influencing the removal efficiency of MPs in the absence of an external coagulant.

As seen in Figure 3a, as the dosage of ACH increased from 30 mg/L to 90 mg/L, the removal efficiency of PET and PE also increased overall. However, when the dosage of ACH was raised to 120 mg/L, the efficiency of PET and PE showed a decreasing trend. This can be attributed to the fact that at low concentrations, the microflocs formed by ACH remain as individual particles. When the concentration was sufficiently high, these microflocs could aggregate into larger masses, thereby boosting coagulation effectiveness [28].

With ACH and FeCl_3_, the maximum removal efficiencies obtained were 91.7 and 98.3%, respectively. In contrast, the values for PET MPs were slightly lower, achieving only 85% and 92%, respectively (Figure 3). The trend is similar to what has been reported in previous studies. Rajala et al. (2020) reported that the highest removal efficiency of 99.4% was obtained with FeCl_3_ and polyaluminium chloride (PACl) coagulants, wherein the metal dosage varied between 0.017 and 1.4 mmol/L, respectively. According to another study [16], the removal efficiency of microfibres resuspended in pure water varied from 86% to 96% based on using optimal PACl concentrations of 4–6 mg/L. In contrast to these two previous studies and this research, [25] reported a relatively low MP removal for <500 µm PE (only 29.70%) by coagulation with domestic wastewater. This could be due to the different mixing conditions and the MP size ranges. The high treatment efficiency in this study could also be attributed to the fact that laundry wastewater forms better flocs compared to municipal wastewater.

### 3.3. Coagulation Mechanism

#### 3.3.1. Charge Neutralization

**Figure 4:**
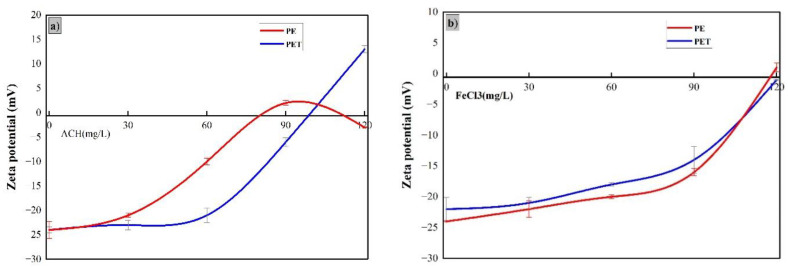
Changes in zeta potential during coagulation: (**a**) laundry waster coagulation with ACH and (**b**) laundry wastewater coagulation with FeCl_3_.

The surface charge of MP particles is negative, and it remains stable in a neutral pH environment [29,30]. Charge neutralization is a key mechanism of coagulation. Hydrolysates from metal coagulants are readily adsorbed onto the surfaces of negatively charged particles, neutralizing their original surface charge and rendering the particles unstable [25]. The zeta potential of the laundry wastewater in the presence of FeCl_3_ and ACH was measured to understand the contrasting behaviours of these coagulants. Figure 4 illustrates the zeta potentials of the MPs used in coagulation experiments and supernatant after settling.

With ACH as the coagulant, the zeta potential of laundry wastewater with high PE and PET crossed the zero point at 90 mg/L and 120 mg/L, respectively (see Figure 4a). The final zeta potential in the PE-ACH system was closer to zero compared to the PET-ACH system, indicating that charge neutralization was more effective in the PE-ACH system. However, in addition to MPs, household laundry wastewater contains a variety of charged components [14]. Indicated here is that charge neutralization alone does not account for the removal efficiencies of PS and PE.

In contrast, FeCl_3_ requires a coagulant dosage of 120 mg/L to achieve zero zeta potential, which facilitates charge neutralization, leading to the destabilization and removal of both PE and PET MPs (see Figure 4b). The charge neutralization in the FeCl_3_-PE system was similar to that in the FeCl_3_-PET system.

In summary, the removal of PET and PE MPs through coagulation with ACH and FeCl_3_ involves charge neutralization. The intensity of charge neutralization varies between different systems, with the PE system exhibiting a stronger charge neutralization effect compared to the PET system.

#### 3.3.2. Adsorption

**Figure 5:**
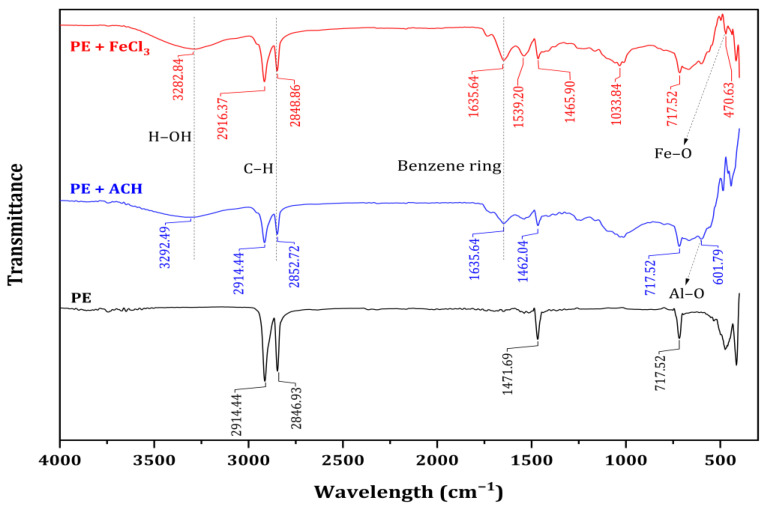
The FTIR spectra of PE microplastics and flocs.

The experimental results show that new bonds were formed during the interaction between PE MPs and coagulants (see FTIR spectra in Figure 5). This indicates that the chemisorption process probably occurred during coagulation.

Besides charge neutralization, adsorption can also play a crucial role in the coagulation process. Theoretically, using aluminium and iron (III) salts as examples, during the hydrolysis of the coagulant and the formation of large flocs, “short-lived” water-soluble aluminium and iron (III) hydroxide complexes are formed. These metal hydroxide complexes also carry a positive charge [31]. This indicates that the amorphous forms of aluminium and iron (III) hydroxide likely had a significant impact on the interaction between coagulants and MPs. According to Zhou et al. [25], the low levels of residual Fe and Al in the water post-coagulation suggest that the majority of the coagulants formed flocs and settled out. Therefore, it is essential to investigate the internal chemical bonds between MPs and flocs to reveal the coagulation mechanism.

When comparing the FTIR spectra of PE before and after coagulation in this study, additional peaks were observed after coagulation (Figure 5). Notably, the prominent peaks around 2915 and 2850 cm^−1^ were attributed to CH_2_ asymmetric and symmetric stretching, respectively [29,32]. The peak around 3435 cm^−1^ was assigned to O-H bond stretching, while the peak at 1729 cm^−1^ was attributed to C=O stretching [33]. The peaks at approximately 1409, 1505, and 1631 cm^−1^ were attributed to substitutions on the benzene ring [33,34].

The FTIR spectra of PE MPs before coagulation and the flocs formed after coagulation are shown in Figure 5. In the FTIR spectrum of the flocs, the peaks at 3292, 2916, and 2852 cm^−1^, which correspond to H-OH and C-H vibrations, were similar to those observed in PE. Additionally, weak absorption peaks at 1635, 1539, and 1466 cm^−1^ indicate the presence of substituted benzene ring groups. The characteristic peaks of PE, located at 717, 2846, and 2914 cm^−1^, remained relatively unchanged after coagulation (see Figure 5).

Compared to the original PE MPs, new peaks appeared in the flocs at approximately 565 cm^−1^ and 620 cm^−1^. These peaks emerged after coagulation with FeCl_3_ and ACH, respectively. The peak at around 565 cm^−1^ is associated with the presence of Fe-O [35], while the peak at around 620 cm^−1^ was derived from the existence of Al-O [36]. Additionally, precipitates were collected from both coagulation tests that used only PAC and FeCl_3_, indicating that new bonds formed during the interaction between PE microplastics and the coagulants. This suggests that a chemisorption process likely occurred during coagulation.

#### 3.3.3. The Change in Solution pH in the Coagulation Process

After adding the detergent, the pH of the washing water only slightly changed from 6.65 ± 0.08 to 6.75 ± 0.05, which could be ignored [19]. In contrast, the coagulation and hydrolysis process significantly alters the pH of the laundry wastewater [37]. When impurities are coagulated in mixtures, their mutual interactions can affect both the coagulant dosage and the optimal pH range. This section examines how the pH varies with different concentrations and types of coagulants.

**Figure 6:**
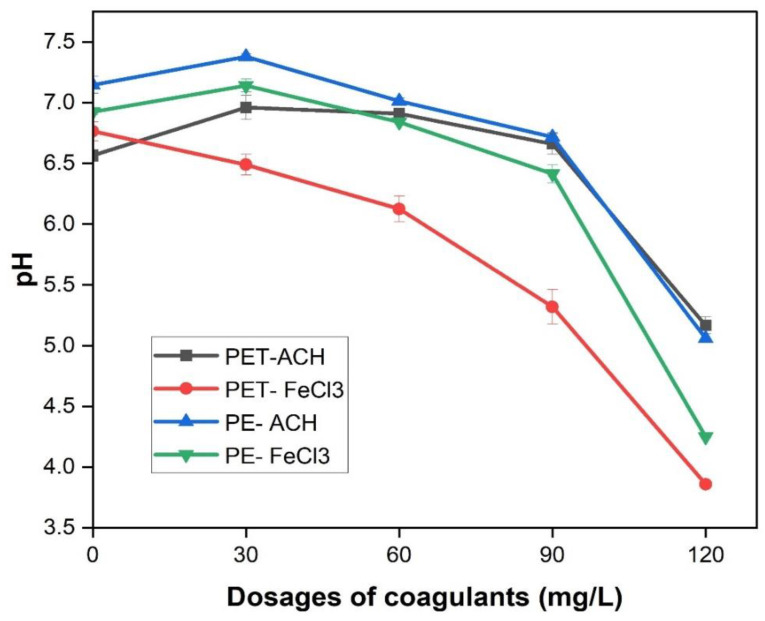
The pH of laundry wastewater with various concentrations and types of coagulant.

The initial pH of domestic laundry wastewater with high MP strength ranged from 6.99 to 7.24. These figures tended to decrease when the Jar Test process began. As can be seen from Figure 6, the pH continues to decrease significantly as the concentration of coagulants increases. The coagulant FeCl_3_ led to a greater reduction in the pH of the laundry wastewater compared to the coagulant ACH. Notably, in laundry wastewater with a high strength of PET MPs, the pH decreased from 6.88 to 3.86 when the FeCl_3_ coagulant was used at a concentration of 120 mg/L. In contrast, the ACH coagulant had less impact on the pH of the laundry wastewater. Specifically, the pH only decreased from 7.0 to 5.1 for laundry wastewater with high strength of PE and PET MPs (see Figure 6**)**. According to a previous study, a small change in the pH of wastewater could lead to a significant impact on the efficiency of the coagulation process [38]. Therefore, ACH, a coagulant that had less impact on the pH, can be prioritized for coagulation in laundry wastewater.

### 3.4. MP Retention by UF Membrane Filtration

Here, the influent sample was the laundry wastewater obtained from the coagulation process at the optimal dosage of the coagulant (90 mg/L for both ACH and FeCl_3_ coagulants). In this study, the 200 kDa UF membrane achieved a 96 ± 2% retention of MPs in quantity, resulting in only 60 MP particles per litre in the UF permeate. These results are similar to what previous studies [39,40,41] documented.

The types of MP removal were also studied. PE was completely retained by UF. The effluent from UF only contained PET fibres, nylon, and rayon. Fibre-shaped MPs were found in the permeate, and they could pass through UF membranes more easily than particle-shaped MPs. This trend is similar to the findings of a prior study. This particular study found that fibres were present in the permeate even after water/wastewater treatment with an RO membrane, which can even retain ions [40].

**Figure 7:**
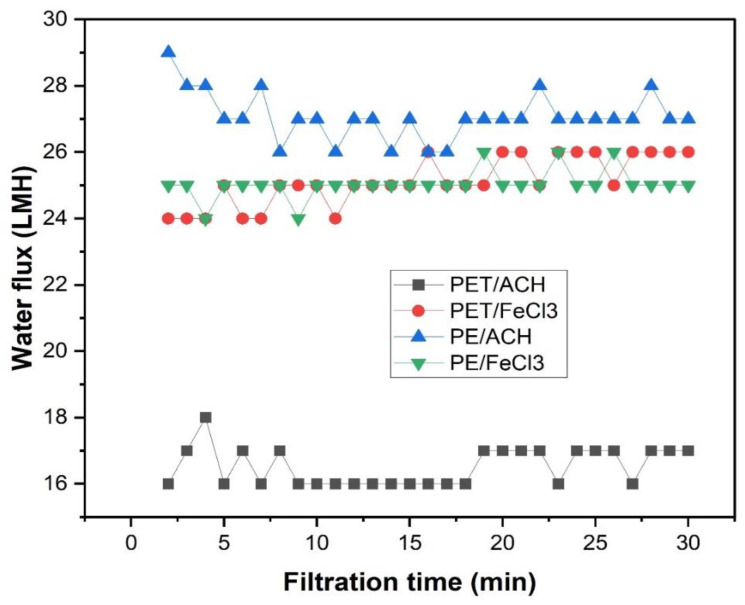
Time-dependent fluxes of the UF membrane.

A major factor restricting the widespread usage of membranes is fouling, which results in significant membrane flux reduction. This will necessitate more cleaning chemicals [40]. Initial experimental results show a promising outcome for all four types of influent samples: laundry water samples with high concentrations of PE and PET, which were pre-treated with ACH and FeCl_3_ coagulants at optimal doses (Figure 7).

As can be seen from Figure 7, there was no flux decline during filtration lasting 30 min. This indicated that fouling did not occur immediately. This could be due to the low concentration of contaminants in the influent, which had been pre-treated with coagulation. Alternatively, it may be because these were preliminary studies, and the filtration time was not long enough to assess the commercial UF membrane’s fouling resistance for laundry wastewater. Thus, in future experiments, test with longer filtration times (24–48 h) should be carried out.

## 4. Conclusions

This study investigates the performance and mechanisms of MP removal from laundry wastewater using a combined approach of coagulation and membrane filtration. Among various coagulants, ACH and FeCl_3_ at a concentration of 90 mg/L demonstrated superior MP removal, with FeCl_3_ slightly outperforming ACH, particularly for PET and PE MPs. Charge neutralization was the dominant removal mechanism during coagulation, with the effect being more pronounced in the PE system than in the PET system. FTIR spectroscopy revealed the formation of new bonds, indicating the emergence of new functional groups during interactions between MPs and coagulants. The pH of the laundry wastewater decreased during coagulation, with FeCl_3_ having a more significant impact compared to ACH. Membrane filtration was successfully used as a polishing MP treatment step to remove fibrous MPs and MPs with low densities, which had not been removed by coagulation. This helped to increase the system’s overall capacity to 99.4%. Short-term experiments indicated that no membrane fouling occurred during the 30 min-filtration process. These findings demonstrate the efficacy of integrating coagulation and membrane filtration for MP removal from laundry wastewater treatment.

## Data Availability

The raw data supporting the conclusions of this article will be made available by the authors on request.
